# Artificial Intelligence–Based Ethical Hacking for Health Information Systems: Simulation Study

**DOI:** 10.2196/41748

**Published:** 2023-04-25

**Authors:** Ying He, Efpraxia Zamani, Iryna Yevseyeva, Cunjin Luo

**Affiliations:** 1 School of Computer Science University of Nottingham Nottingham United Kingdom; 2 Information School University of Sheffield Sheffield United Kingdom; 3 School of Computer Science and Informatics De Montfort University Leicester United Kingdom; 4 School of Computer Science and Electronic Engineering University of Essex Colchester United Kingdom; 5 Key Laboratory of Medical Electrophysiology, Ministry of Education & Medical Electrophysiological Key Laboratory of Sichuan Province, Collaborative Innovation Center for Prevention of Cardiovascular Diseases, Institute of Cardiovascular Research Southwest Medical University Luzhou China

**Keywords:** health information system, HIS, ethical hacking, open-source electronic medical record, OpenEMR, artificial intelligence, AI-based hacking, cyber defense solutions

## Abstract

**Background:**

Health information systems (HISs) are continuously targeted by hackers, who aim to bring down critical health infrastructure. This study was motivated by recent attacks on health care organizations that have resulted in the compromise of sensitive data held in HISs. Existing research on cybersecurity in the health care domain places an imbalanced focus on protecting medical devices and data. There is a lack of a systematic way to investigate how attackers may breach an HIS and access health care records.

**Objective:**

This study aimed to provide new insights into HIS cybersecurity protection. We propose a systematic, novel, and optimized (artificial intelligence–based) ethical hacking method tailored specifically for HISs, and we compared it with the traditional unoptimized ethical hacking method. This allows researchers and practitioners to identify the points and attack pathways of possible penetration attacks on the HIS more efficiently.

**Methods:**

In this study, we propose a novel methodological approach to ethical hacking in HISs. We implemented ethical hacking using both optimized and unoptimized methods in an experimental setting. Specifically, we set up an HIS simulation environment by implementing the open-source electronic medical record (OpenEMR) system and followed the National Institute of Standards and Technology’s ethical hacking framework to launch the attacks. In the experiment, we launched 50 rounds of attacks using both unoptimized and optimized ethical hacking methods.

**Results:**

Ethical hacking was successfully conducted using both optimized and unoptimized methods. The results show that the optimized ethical hacking method outperforms the unoptimized method in terms of average time used, the average success rate of exploit, the number of exploits launched, and the number of successful exploits. We were able to identify the successful attack paths and exploits that are related to remote code execution, cross-site request forgery, improper authentication, vulnerability in the Oracle Business Intelligence Publisher, an elevation of privilege vulnerability (in MediaTek), and remote access backdoor (in the web graphical user interface for the Linux Virtual Server).

**Conclusions:**

This research demonstrates systematic ethical hacking against an HIS using optimized and unoptimized methods, together with a set of penetration testing tools to identify exploits and combining them to perform ethical hacking. The findings contribute to the HIS literature, ethical hacking methodology, and mainstream artificial intelligence–based ethical hacking methods because they address some key weaknesses of these research fields. These findings also have great significance for the health care sector, as OpenEMR is widely adopted by health care organizations. Our findings offer novel insights for the protection of HISs and allow researchers to conduct further research in the HIS cybersecurity domain.

## Introduction

### Context

The health care sector is continuously targeted by cyberattackers, who seek to exploit undetected vulnerabilities in critical health infrastructure. Such attacks can cause service disruptions, financial losses, and harm to patients. In the 2017 WannaCry attack on the United Kingdom’s National Health Service (NHS), there was a substantial decrease in patients’ attendances and admissions numbers, which caused a £5.9 million (US $7.1 million) lost in terms of hospital activity [1]. This study is motivated by recent security incidents that have increased during the COVID-19 pandemic, affecting health care organizations, such as the US Department of Health and Human Services, the World Health Organization (WHO), and pharmaceutical companies [2]. Specifically, the United States Public Health Service reported that approximately 100 million pieces of patient information were stolen monthly by 2020 [3]. Fortified Health Security, a leading organization in health care cybersecurity, reported that more than 400 health information system (HIS) providers had been breached, affecting approximately 13.5 million patients [4]. In such cases, cyberattackers not only destroy the HIS but also gain access to and can modify sensitive health records that may mislead medical diagnosis [5].

The research community and health care industry have long realized the urgency to protect HISs [6-12]. However, existing cybersecurity research in the health care domain places an imbalanced focus on protecting medical devices [13-17] and medical data [18], whereas previous studies do not offer a systematic approach for the investigation of HIS breaches or for improving cybersecurity more broadly. In this study, we propose a systematic approach to address this shortcoming based on ethical hacking. Typically, ethical hacking entails analyzing a system to identify potential weak points and then executing attacks to test the robustness of the system. Such approaches often entail using artificial intelligence (AI) and, most typically, reinforcement learning, for example [19]. However, reinforcement learning has important shortcomings when it comes to the ethical hacking of HISs, namely, reinforcement learning requires large data sets for training purposes, which most often are unavailable. Therefore, as an approach, it can be unreliable [20]; can cause severe issues for the HIS network [21]; and requires skills and expertise, neither of which are widely available [22].

### Objectives

In our study, we address the above limitations by proposing a new optimization module for ethical hacking that uses the ant colony optimization (ACO) algorithm. The algorithm is characterized by positive feedback, distributed computation, and constructive greedy heuristics [23]. ACO has been previously implemented in the cybersecurity domain, focusing on network intrusion detection, and has recently been proposed for vulnerability analysis and detection [24].

In this study, we built an HIS simulation platform by implementing an open-source electronic medical record (OpenEMR) system and drew from the ethical hacking framework from the National Institute of Standards and Technology (NIST), which we enriched by integrating ACO within its optimization module as part of our ethical hacking method to examine the exploitation of potential vulnerabilities of HISs. We then demonstrated ethical hacking for the HIS simulation environment using both optimized and unoptimized hacking methods and compared the results.

Our study makes important contributions to the health care industry from a cybersecurity perspective. First, our methodological approach to ethical hacking provides important insights into the protection of HISs. It allows practitioners to identify potential vulnerabilities in their systems and offers researchers several avenues for future research. Second, our optimized ethical hacking approach addresses the weaknesses of preexisting frameworks by proposing an intelligent and maintainable ethical hacking solution. To the best of our knowledge, there is no systematic AI-based ethical hacking method that is tailored for health care organizations. Our research makes a major theoretical and practical contribution to the field of digital health by addressing the security aspects of digital medicine infrastructure, which will ultimately improve the quality of security practices of large health care organizations. In doing so, our findings indirectly inform cognate disciplines, namely information systems literature and cybersecurity, by being centered on a core information system element [25].

### Background

#### HIS Security

New technologies have been advancing the field of HISs and improving the quality of services in the health care sector [26-28]. Some advanced HISs support medical diagnoses based on existing health records and data gathered from intelligent medical devices. Such systems significantly reduce the workload of health care professionals and enable early detection, diagnosis, and intervention, thereby increasing the success rate of treatment [29,30]. However, new technologies introduce new security risks for HISs, and the lack of sufficient security control is a concern [31]. According to recent studies, HISs have major security vulnerabilities [32-34] and privacy concerns [35]. For example, access to insecure web pages and default coded passwords are common vulnerabilities introduced by medical devices [36]. Similarly, insecure communications on unauthorized and unencrypted web services are also common vulnerabilities because they allow cyberattackers to gain remote access to HISs [37].

As a result, to date, most studies in the health care cybersecurity domain have focused primarily on increasing the security of medical devices [13-17] and the protection of medical data [18]. For example, a common approach is to implement data encryption mechanisms [13], often in combination with scrambling techniques [18], to protect wavelet-based electrocardiogram (ECG) data both in transit and storage. Other popular solutions involve the design and use of access control schemes to further increase the protection of shared health data [14], implementation of authentication protocols for wearable devices [15], and adoption of privacy-aware profile management approaches that help manage the privacy of patient electronic profiles [14]. In other cases, the proposed solutions involve mechanisms that enhance heartbeat-based security [17]. However, existing research has not yet offered a systematic approach or methods to investigate and understand how attackers can breach HISs and access health care records. To address this, we discuss the ethical hacking methods that have been proposed by cybersecurity research, which can provide a systematic approach.

#### Ethical Hacking Methods

Some of the most widely adopted ethical hacking methods are the NIST framework [38], Penetration Testing Execution Standard (PTES), and framework proposed by the Open Web Application Security Project (OWASP). In addition, different organizations often develop their own organization-specific methods that correspond to their particular organizational needs [22].

Both ethical hacking and penetration testing are authorized attempts to gain unauthorized access to computer systems or data. Penetration testing is a subset of the ethical hacking methods. Penetration testing assesses a specific aspect of a system that is usually restricted by an outlined scope, whereas ethical hacking has more flexibility without being restricted [39]. However, systematic ethical hacking or penetration testing typically includes 4 main modules: information gathering, discovery, attacking, and reporting. The tester performs a reconnaissance at the information-gathering stage and collects information about the target HIS. At the discovery stage, the tester attempts to understand the system’s structure of the system and analyze its paths and directories. Next, the tester identifies the vector to attack at the attack stage, which is typically based on the vulnerability scanner results. Finally, at the reporting stage, the tester uses all evidence gathered during the previous stages to prepare a report documenting major findings.

The extent to which such ethical hacking methods will be successful largely depends on the skills and expertise of professional testers involved in penetration testing. However, the number of skilled programmers in cybersecurity, particularly in the health care domain, is limited [22]. This means that on the one hand, it is difficult to identify the necessary talent for ethical hacking within such complex environments, whereas on the other hand, there is a risk of poorer performance when the required skills are not available.

#### Ethical Hacking Tools and Solutions

Nettacker, a solution developed by OWASP, contains an optimization module, but it is not as mature, not fully published, and lacks an exploit module. This means that a given user will have to select the exploit tools and payload on their own, which can be challenging for nonexperts in cybersecurity. APT2, the solution offered by the Massachusetts Institute of Technology, uses Network Mapper (Nmap) to scan information. An exploit can be launched from its library, depending on the scanning information, and it has a knowledge base that can record the information of the targeted host. Nevertheless, it lacks an optimization module. This finding suggests that the accuracy and efficiency of ethical hacking risks are inferior. Similar to APT2, Autosploit [40], a solution that combines Shodan, Censys, Zoomeye, and Metasploit, does not have an optimization module. It is easy to conduct ethical hacking using this solution because it requires only logging into a Shodan account and provides details regarding the targeted host. After performing a search, Shodan will provide the open port, the vulnerabilities that exist, and tools for the exploit, which will then be able to input this information to Metasploit, specifying the local host and the local port [41]. Metasploit can then run the exploit automatically. However, similar to APT2, Autosploit risks have less accuracy and efficacy because it cannot be optimized. Currently, it is unfeasible to test all possible system configurations. An earlier study attempted to address this problem and proposed the use of generalized binary splitting and the Barinel method to optimize the efficiency of Autosploit [40]. Although this approach positively influenced Autosploit’s performance, the tool library and database of vulnerabilities stopped being updated in 2019 and are now outdated.

#### AI-Based Ethical Hacking in HISs

Ethical hacking methods often use AI techniques. Among those most often used is reinforcement learning, which helps identify and analyze vulnerabilities in information systems. To date, reinforcement learning has been successfully applied in simulated environments to analyze vulnerabilities using the Partially Observed Markov Decision Process [42] and within the context of applied Q-learning with a deep neural network architecture [19]. However, these approaches tend to offer mostly theoretical insights and are being implemented in MATLAB; to date, they have not been systematically integrated into any ethical hacking method. Another major shortcoming is that reinforcement *learning* requires a vast amount of data and ample time to train the model. In reality, it is unlikely that a single targeted host will exhibit sufficient vulnerabilities to train the algorithm. Additionally, reinforced learning can be unreliable for ethical hacking. For example, it has been used in the past for learning control policies in Atari games, whereby an agent triggers several bugs to achieve a high score; however, such behavior does not form part of the ethical hacking plan [21] and causes severe problems for the whole network, which is undesirable. Finally, most importantly, reinforcement learning is characterized by low reproducibility because of its data requirements and because its results can be negatively affected by even small environmental changes such as machine upgrades [20].

#### ACO Approach

In this paper, we propose the use of the ACO approach as an optimization algorithm to enhance the optimization module for ethical hacking. This algorithm is characterized by positive feedback, distributed computation, and constructive greedy heuristics [23] and can be particularly beneficial during attack path analysis, which is the core part of ethical hacking optimization.

ACO is an evolutionary algorithm often used to solve various optimization problems, for example, the traveling salesman problem (TSP). Optimization problems such as the TSP are particularly relevant to identifying and analyzing attack paths as part of ethical hacking, as in both cases, the objective is to construct the shortest path between the point of origin and the target point. In more detail, the goal of the TSP is to identify the shortest or quickest path for a salesman to arrive at their destination while covering all nodes between the point of origin and the target point and visiting them only once. Similarly, in ethical hacking, the goal is to attack the targeted machine by investigating some already known vulnerabilities and their exploitation (exploits) that can be combined to complete the attack successfully.

To date, the ACO approach has been implemented in the cybersecurity domain, focusing on network intrusion detection, which is a passive form of defense. More recently, it was proposed to be efficient for vulnerability analysis and detection, informed by bioinspired cybersecurity research [24]. On the basis of these earlier findings, our study integrated ACO within the optimization module of ethical hacking to examine its performance regarding the exploitation of potential vulnerabilities of HISs.

## Methods

### Simulation Platform

For the purposes of our study, we set up a virtual environment to avoid acting directly in a real-world setting, thus causing potential damage to the HIS. Specifically, we designed an experiment to simulate an HIS.

#### Targeted Host and Attack Host

In ethical hacking, the targeted host machine is attacked by the host machine. We installed the Kali Linus System 2021.1 on a virtual machine workstation in our simulation environment, which acts as the attack host. In addition, we installed Ubuntu 20.04.2.0 on another virtual machine workstation, which acted as the targeted host. Table 1 summarizes the hardware details of the target and attack hosts. Information on the software and services of the targeted host that simulates a medical worker is presented in Table 2.

As part of our experiment, we adapted the NIST ethical hacking framework [38] and follow the core planning, discovery, attack, and reporting modules. We first set up a simulation environment by implementing an OpenEMR system and then launched ethical hacking to exploit the vulnerabilities of the simulated HIS.

**Table 1:** Hardware details for the targeted machine and attack machine.

	Target host	Attack host
Location	VM^a^ workstation	VM workstation
System	Ubuntu 20.04.2.0	Kali Linux system 2021.1
Kernel	Linux version 5.8.0-59-generic	Linux version 5.8.0-59-generic
Memory	4 GB	4 GB
Bandwidth	100 Mbps^b^	100 Mbps
Hard disk space	2 GB	20 GB
Core of CPU^c^	8	4
Kind of CPU	Intel core i7-9750 CPU 2.6 GHz	Intel core i7-9750 CPU 2.6 GHz

^a^VM: virtual machine.

^b^Mbps: megabits per second.

^c^CPU: central processing unit.

**Table 2:** Software and services used on the targeted machine.

	Version	Description
PHP	PHP v8.1.0	PHP is a hypertext preprocessor, ie, a scripting language on a server, which is used by OpenEMR^a^.
Apache2	Apache v2.0	Apache is the most popular web server software for building a website. In the targeted host, it is used by OpenEMR.
MySQL	MySQL v5.7.17	MySQL is one of the most popular relational database management systems. It has a small volume, high speed, and low maintainable cost, which is used by OpenEMR.
MySQL	MySQL v5.7.17	MySQL is one of the most popular relational database management systems. It has a small volume, high speed, and low maintainable cost, which is used by OpenEMR.
OpenEMR	OpenEMR v6.0.0	OpenEMR is an open-source electronic medical record system. In the targeted host, it is used to simulate a medical worker’s machine.
Vsftp	Vsftp v3.0.3	Vsftp provides a designedly open port installed for the experiment environment. It has many dangerous vulnerabilities.
OpenSSH-server	OpenSSH-server v1.8.2	OpenSSH-server provides a designedly open port installed in the experiment environment. It has many dangerous vulnerabilities.

^a^OpenEMR: open-source electronic medical record.

#### OpenEMR Implementation

In our HIS simulation platform, we implemented OpenEMR. Overall, OpenEMR is a complex system with key functionalities, including practice management, EMR management, scheduling, electronic billing, prescribing, a patient portal, and a clinical decision support system, and has a complex database of more than 100 tables. We purposefully chose to implement this HIS because it supports a comprehensive security risk-management scheme based on the Health Insurance Portability and Accountability Act and NIST standards [43]. In addition, it is certified by the Office of the National Coordinator for Health Information Technology, which can run on different platforms such as Windows, Linux, and Mac OS X, and it is the most widely adopted HIS [44].

### AI-Based Ethical Hacking Method

#### Overview

Our adaptation of the NIST ethical hacking framework [38] consisted of following 6 modules: scanning, discovery, exploitation, optimization, reporting, and control. In other words, we used the original NIST modules, but further enhanced them with 2 additional modules: optimization and control. Table 3 summarizes the key activities of each stage.

We conducted a comparative experiment between AI-based and non–AI-based ethical hacking methods. Although the AI-based experiment followed the 6 stages of the ethical hacking method as indicated above, the non–AI-based experiment followed the same method without executing the optimization module. Optimized and unoptimized penetration tests were performed 50 times to reduce the uncertainty caused by the simulation environment. In each run, information on the time, the number of exploits, and the number of successful exploits were recorded and compared.

**Figure 1:**
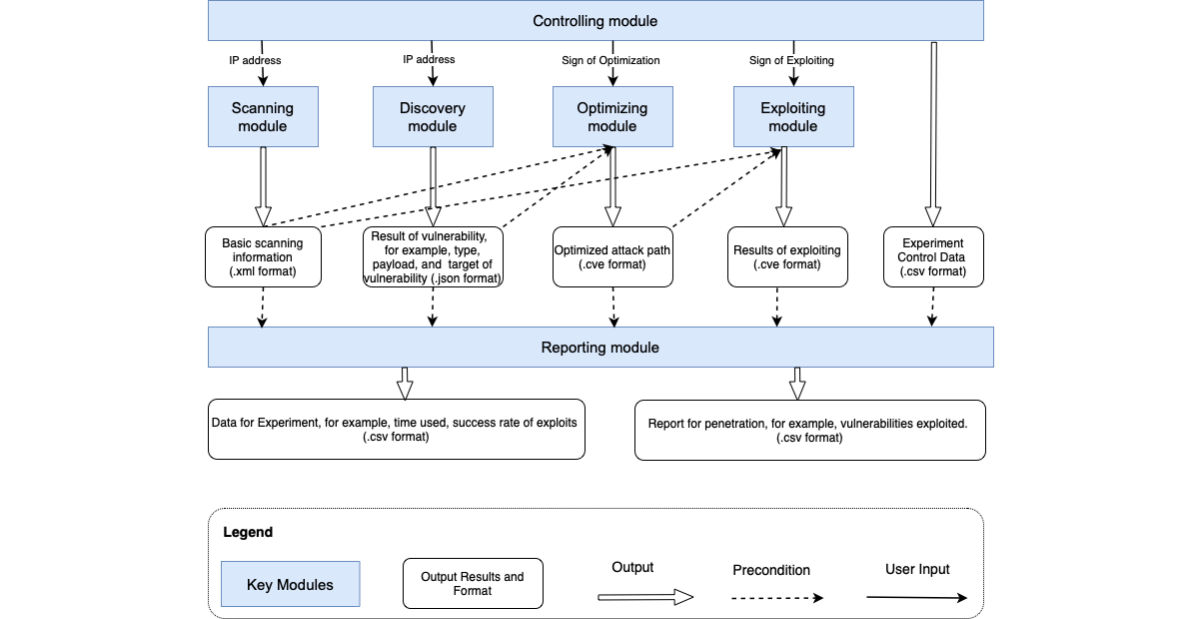
Interactions between different modules.

Generally, the results from each module were first recorded and then used in each subsequent module. Figure 1 shows the interactions between different modules and the results from each module.

**Table 3:** Key activities and the National Institute of Standards and Technology (NIST) method coverage of the (artificial intelligence [AI]–based) ethical hacking method.

(AI-based) ethical hacking stages	Key activities	NIST method stages
Scanning	Use the Nmap^a^ scanning tool to identify the number of ports, port status, protocol, and operating system	Planning discovery
Discovery	Use the Xray scanning tool to identify vulnerabilities	Planning discovery
Exploiting	Use attacking tools (eg, SQLMap and Metasploit) to probe networks, applications, and database-related flaws and vulnerabilities	Attack
Optimizing (optional)	Optimize attack paths using AI (eg, ACO^b^)	Discovery attack
Controlling	Coordinate the modules to launch attacks and set ethical hacking preferences	Planning discovery attack
Reporting	Collect and report results on the exploit	Reporting

^a^Nmap: Network Mapper.

^b^ACO: ant colony optimization.

#### Scanning Module

As part of the scanning module, we scanned the host information of the targeted machine, including the port, operating system, and installed service of the targeted machine. Nmap was used as a scanning tool to collect this information. Other similar tools included ZMap and Masscan. ZMap has an accuracy rate similar to that of NMap, but its computational time is higher [40]. Masscan is faster, but its accuracy rate is lower, particularly when the scanning area increases [40]. Therefore, we selected Nmap because of its accuracy and efficiency (computational time) and because it has more than 200 extension scripts for scanning.

We developed the following 2 versions of Nmap scanning scripts: the first was used for a single IP address and the other was used for an IP address segment. For a single IP address, Nmap scanning imports the IP from the control modules, checks whether the host is alive, and then scans and reports the results. For an IP address segment, the tool adopts multithreading to support multiple IP addresses in an IP address segment, and, as in the previous case, it then scans and reports the results.

#### Discovery Module

This module focuses on obtaining vulnerability-related information of the target host. Existing vulnerability scanning tools include Nessus, NexSpose, and Xray. Although Nessus and Nexpose have a Metasploit application programming interface, and their vulnerability data set is one of the largest for vulnerability scanning, they are costly, and the education version has a limited number of vulnerabilities and ports.

In this study, Xray was selected for vulnerability scanning using the basic crawler method. Xray is a free vulnerability scanning tool, and their performance is comparable to that of Nessus and Nexpose. Xray supports diverse operating systems such as Windows, Linux, and Mac. As a passive scanning tool, it is much faster than active scanning because the latter requires sending requests to the targeted host and waiting for a response. Passive scanning is also challenging to detect using a targeted host. Xray also supports the use of web scanning. The Xray output is a JSON file that contains the type, payload, and target of the vulnerability. Because the targeted machine is an HIS using OpenEMR, the web scanning module can help detect vulnerabilities in OpenEMR.

#### Exploiting Module

This module launches attacks on the targeted host by leveraging the information gathered in the previous modules. This module applies ethical hacking tools, namely, SQLMap and Metasploit. Many exploiting tools provide similar performance and functionalities; however, we selected Metasploit as the primary attack tool because it is the most powerful and widely used tool in the field. This tool integrates several application programming interfaces that can be used for manual and automated exploitation using predefined settings. When conducting a manual penetration test, the tester must set up the targeted information and tools used for exploitation. The exploitation procedure is replaced by a resource scripts file that configures the Metasploit when using automated scripts. In our study, we imported output files from Nmap and Xray, ran automated exploits, and extracted the exploitation results.

In addition, as the database is an essential component of the HIS, attacks should be launched as part of ethical hacking, and the vulnerabilities of the database should be exploited. For this purpose, we used SQLMap to conduct attacks on a database that launches attacks by executing malicious SQL commands in the web input. It supports 5 types of SQL injections and can launch other types of exploits, such as XSS (cross-site scripting) injection [45]. By exploiting database vulnerabilities using SQLMap, the attacker can tamper with or steal digital data and information, remotely control the database, crash the hard disk, and control the system using Trojan viruses [46]. However, this behavior does not damage the targeted host, which is essential because the penetration test aims to enhance security rather than destroy the system. In our experiment, SQLMap imported the JSON output file from Xray and retrieved the URL for SQL injection. It then launched the attack automatically and exported a file using exploitation results.

#### Optimizing Module

For the optimizing module, we used ACO as the optimization algorithm for the optimization module. ACO simulates the behavior of ants to identify the shortest path(s) and pheromone-based communication within the colony. Attack path analysis is a core aspect of ethical hacking optimization. In ethical hacking, the goal is to attack the targeted machine using known paths, and the objective is to identify the shortest or fastest path to achieve this. The most common example of using ACO is to solve the TSP, where the shortest or fastest path is searched for by a salesman to deliver goods in all cities by exploring various paths and visiting each city exactly once. Ethical hacking has a similar goal, whereby the objective is to attack the targeted machine by exploiting as few known vulnerabilities as possible to successfully and swiftly complete the attack. Various paths between the origin and target machines can be built by combining exploits and finding the shortest or fastest way to do so. Textbox 1 demonstrates the optimization procedure for ACO.

The optimization module reads the file (“ant_cve.json”) as the input, which contains the history information of the exploits of the targeted host. Common vulnerabilities and exposures were allocated to different nodes of the vulnerability matrix. The path represents the set of successful exploits selected out of all the launched exploits, the ant represents a potential solution, and a path composed of a set of exploits is described as the payload. The concentration at each node depends on the severity level of the identified vulnerability. Here, each ant probes for building a path by combining nodes of the path into a successful trial and informs other ants on the results of such an attempt by sharing certain information, such as the intensity of the trail between 2 nodes *i* and *j* at some moment of time *t*, denoted as 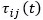 and visibility 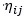


ACO starts with initialization, where the number of ants (m=40) is selected, and the number of iterations is set to 50. Initially, all the ants were positioned in different nodes of the vulnerability matrix. The intensity of the trail between each pair of nodes *i* and *j* at the initial moment of time *0* was set to a small constant 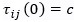. The pheromone concentration 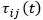 is updated after each iteration of each path as follows:



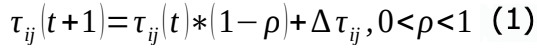



where *ρ* is the volatilization of pheromone, and it refers to reductions in the pheromone after each run, which is set to *ρ*=0.3, according to Axinte [46], and 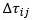 is computed as follows:



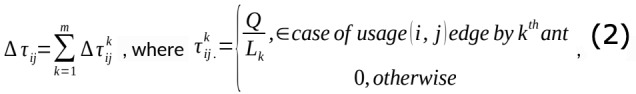



where *Q* is a constant and *L_k_* is the length of the *k*th ant tour.

The visibility for a pair of 2 node is computed as 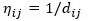 and is an inverse of the Euclidian distance between them. The global best and shortest path value was computed as the distance between the origin and target. It was initially set to 9999, and the evolution process was started by updating it to any real distance value after the computation of the first path at the end of the first iteration.

The transition probability for each pair of nodes *i* and *j* for the *k*th ant can then be computed as follows:



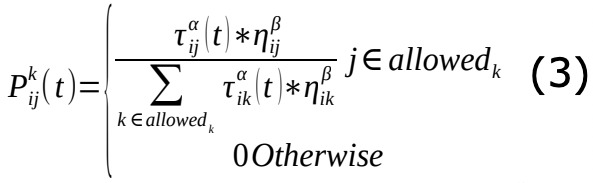



where *allowed* is the set of not yet visited nodes, α is the weight of the pheromone, and β is the weight of the heuristic value [23]; here, they are set to α=.7 and β=.7, as suggested in Liu et al [47].

Each successful use of an exploit increases the concentration of pheromones for a pair of exploits that are connected successfully.

An ant explores a set of nodes vulnerabilities presented in a matrix in an attempt to construct a successful exploitation path. Whenever a successful exploitation is recorded, the current successful path is compared with the global best path found in all runs thus far and updated every time a shorter path is found.

The end condition for each literation was whether all the ants visited all the nodes in the vulnerability matrix. When all iterations have finished, ACO ends and provides results on the global best path.

After the final iteration, the optimization module reports the list of attack paths and prioritizes the paths with the highest pheromone concentration. The output was then stored as a *.csv file, titled “ant_output.csv,” which contains information on the common vulnerabilities and exposures, exploit, and used payload.

**Algorithm 1 (the ant colony optimization [ACO] algorithm: ACO(Num_Iters, Num_Ants, VulnerList).**

Require:

NumIters (NumIters >0) # the maximum number of iterations,

NumAnts (NumAnts >0) # the maximum number of ants,

VulnerList # the vulnerability exploits list.

Ensure

The best path (BestPath) is exported.

1: BestPath ← 0; BestPathDist ← 99999999;

2: For k← 1: NumIters do

3: LocalBestPath ← 0; LocalBestPathDist ← 99999999; # local best path for a single iteration

4: PheromCons ← zeros([][]); # matrix of pheromone concentrations for all pairs of ants.

5: For i← 1: NumAnts do

6: Vulner_i = VulnerList[i];

7: For j← 1: NumAnts do

8: Vulner_j = VulnerList[j];

9: p_ij=compute_Pij(Vulner_i, Vulner_j); # transition probability for pair (i,j).

10: CurrentPath_ij=computeProbablePath(p_ij, Vulner_i, Vulner_j); # path for pair (i,j)

11: CurrentPathDist=computerPathDist(CurrentPath_ij); # distance for path (i,j).

12: PheromCons(i,j) = updatePheromCons(CurrentPath_ij); # update of pheromon matrix

13: If (CurrentPathDist<LocalBestPathDist)

14: LocalBestPath=CurrentPath; # update of the shortest local path

15: LocalPathDist=CurrentPathDist; # update of the shortest local path distance

16: End if

17: End for #NumAnts with j index

18: End for #NumAnts with i index

19: If (LocalBestPathDist<BestPathDist)

20: BestPath=LocalBestPath; # update of the shortest global path

21: BestPathDist=LocalBestPathDist; # update of the shortest global path distance

22: End if

23: End for #NumIters

24: return BestPath

#### Controlling Module

The controlling module imports the results produced from the previous modules, and it is necessary to conduct ethical hacking and launch attacks. Users can control the penetration test via an interactive user interface and set the targeted machine’s IP address or IP address segment of the targeted machine. The module then transmits this information to the information and vulnerability scanning modules. Once the scanning module is completed, users have to decide whether optimization is needed, and based on their decision, the optimization module will be triggered. This, in turn, calls the exploiting module to launch the attack on the targeted host. At the end of the procedure, this module sends its results to the reporting module, recording the time required to carry out ethical hacking for each module.

#### Reporting Module

**Figure 2:**
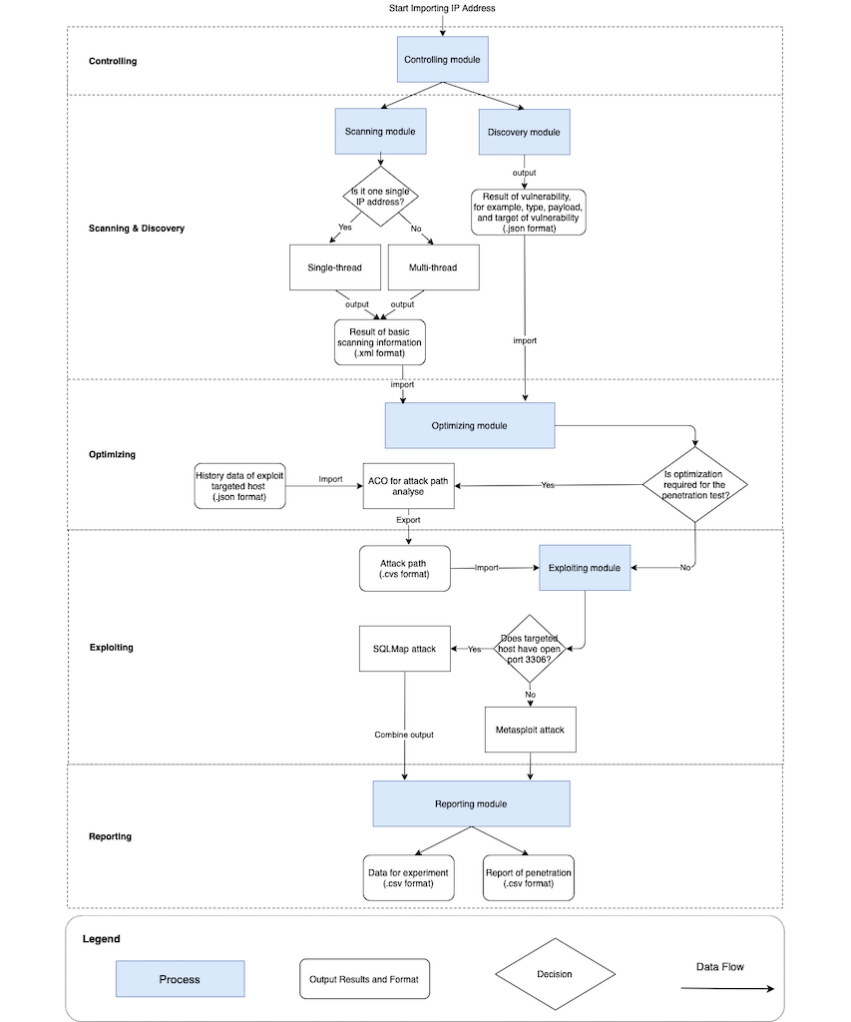
Flowchart of the ethical hacking framework. ACO: ant colony optimization.

The reporting module collected the results of ethical hacking. Two sets of results (.csv files) were generated. The first set reports the time used for each module and the number and success rate of the launched exploits. This information can also be used to evaluate the performance of the algorithm in the optimization module. The second set of results contains information regarding the vulnerabilities themselves and can help users understand the targeted host’s security status and, therefore, act accordingly. Figure 2 summarizes the execution of the ethical hacking framework.

### Ethical Considerations

As our research does not involve human participants directly or indirectly (eg, observations of public behaviors or secondary analyses of research data), ethics approval, informed consent, and compensation for human participants research were not required. In addition, the design of our study was based on simulations conducted within an experimental setting; as such, it did not raise any privacy or confidentiality concerns.

## Results

We performed AI-based (optimized) and non–AI-based (unoptimized) ethical hacking on the target machine (host IP 192.168.1.44). The AI-based experiment followed the novel ethical hacking framework (see the *Methods* section). The non–AI-based experiment followed the same method but omitted the optimization module. Table 4 shows the key activities across the different modules according to the proposed 6-stage ethical hacking method.

Both the optimized and the unoptimized ethical hacking were run 50 times (50 runs) each to account for the stochastic nature of ACO and to reduce the uncertainty owing to the simulation environment. The information regarding execution time, the number of exploits investigated, and the number of successful exploits used to construct the attack path was recorded for each run.

Table 5 presents the results of 50 runs of comparison of unoptimized and optimized ethical hacking methods, where the average time used to perform the penetration test, the success rate of all penetration tests, and the highest and average rates of exploits with regard to all exploits were used as comparison metrics. The highest numbers of exploits were 11 and 20, the average numbers of launched exploits were 8 and 14, the numbers of successful penetration tests were 32 and 42, the numbers the highest number of successful exploits were 9 and 18, and the average numbers of successful exploits were 5 and 11 for the unoptimized and optimized ethical hacking methods, respectively.

**Figure 3:**
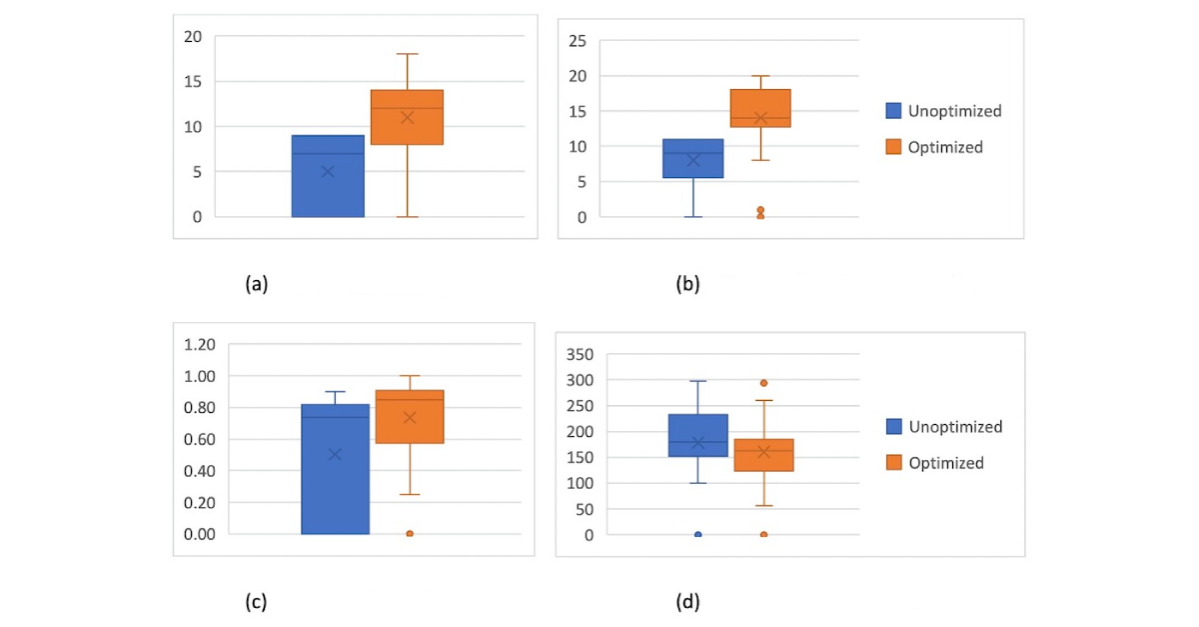
Results of the computational experiments for both unoptimized and optimized ethical hacking methods. (A) Total number of exploits; (B) Number of successful exploits; (C) Success rate results; (D) Average execution time.

Figure 3 depicts in a box plot (each box composed by quartiles 1-3) the total number of launched exploits (Figure 3A) and successful exploits (Figure 3B) for both optimized and unoptimized ethical hacking methods with the average (indicated by X), median (indicated by straight line across the box), and SD (indicated by whiskers, which might go outside of the box plot or overlap with it). Figure 3C depicts the box plots of the rate of successful exploits with respect to the total number of exploits, and Figure 3D shows the average execution time for both optimized and unoptimized ethical hacking methods.

To show an example of the results in a single run, the last run out of 50 runs for the unoptimized and optimized ethical hacking methods were compared for the penetration test for 192.168.1.44. The results of the unoptimized method show that the method ran for 177 seconds; out of 9 exploits, 7 were successful; and these exploits were related to improper input validation, cross-site request forgery, remote code execution (in Windows Remote Desktop Gateway), denial of service attacks, improper authentication, remote access backdoors, and the deserialization of untrusted data. In the case of the optimized method, the method ran for 153 seconds, and only 6 exploits were investigated, all of which were used to build a successful attack path.

The details of the exploits used for building a successful path are presented in Table 6, which are related to remote code execution, cross-site request forgery, improper authentication, vulnerability in the Oracle Business Intelligence Publisher, an elevation of privilege vulnerability (in MediaTek), and remote access backdoor (in the web graphical user interface for the Linux Virtual Server).

**Table 4:** Key activities for the experiment setting for each of the 2 ethical hacking the methods section.

Module	Key activities	Optimized	Unoptimized
Scanning	Use the Nmap^a^ scanning tool to identify the number of ports, the port status, protocol, and operating system	Yes	Yes
Discovery	Use the Xray scanning tool to identify vulnerabilities	Yes	Yes
Exploiting	Use the SQLMap tool to exploit SQL injection related vulnerabilities Use the Metasploit tool to probe networks and applications related flaws and vulnerabilities	Yes	Yes
Optimizing (optional)	Optimize the attack path using ACO^b^	Yes	No
Controlling	Use results from the modules above to launch attacks Provide an interactive interface that allows users to specify the IP of the targeted machine	Yes	Yes
Reporting	Collect and report results on vulnerabilities, time used, and the success rate of the launched exploits	Yes	Yes

^a^Nmap: Network Mapper.

^b^ACO: ant colony optimization.

**Table 5:** Comparison of the results of optimized and unoptimized ethical hacking after 50 runs.

Metrics	Unoptimized	Optimized
Average used time (in seconds)	178	160
Success rate of all penetration tests (%)	64	98
Highest success rate of exploit or exploits (%)	88.9	100
Average success rate of exploit or exploits (%)	50.3	73.5

**Table 6:** Exploits used in the successful attack path found by optimized ethical hacking.

	Exploit	CVE^a^ number	Details
0	V7	CVE-2020-0610	/exploit/windows/browser/adobe_flash_otf_font
1	V8	CVE-2012-0714	/exploit/windows/browser/adobe_flash_regex_value
2	V9	CVE-2012-5975	/exploit/linux/http/piranha_passwd_exec
3	V17	CVE-2021-23919	/exploit/unix/webapp/openemr_upload_exec
4	V4	CVE-2017-0503	/exploit/windows/browser/adobe_flash_otf_font
5	V2	CVE-2000-0248	/exploit/linux/http/symantec_web_gateway_lfi

^a^CVE: common vulnerabilities and exposures

## Discussion

### Brief Summary of Findings

In this study, we propose a novel methodological approach to ethical hacking in HISs. We conducted a comparable experiment by launching ethical hacking using both the optimized and unoptimized methods. In particular, we set up an HIS simulation environment by implementing the OpenEMR system and followed the NIST ethical hacking framework to perform ethical hacking. We launched 50 rounds of attacks using both the unoptimized and optimized methods. The results show that the optimized ethical hacking method outperforms the unoptimized method in terms of average time used, the average success rate of exploitation, the number of exploits launched, and the number of successful exploits. We were able to identify the successful attack paths and exploits that are related to remote code execution, cross-site request forgery, improper authentication, vulnerability in the Oracle Business Intelligence Publisher, an elevation of privilege vulnerability (in MediaTek), and remote access backdoor (in the web graphical user interface for the Linux Virtual Server). Theoretically, these findings contribute to HISs, ethical hacking methodology, and mainstream AI-based ethical hacking methods. Practically, the findings have great significance for the health care sector, specifically because OpenEMR is widely adopted by health care organizations.

### Implications

Our work contributes to the HIS security domain by proposing an AI-based method for ethical hacking that helps identify vulnerabilities in HISs. In particular, we set up a simulation environment by implementing OpenEMR and performed systematic ethical hacking on this virtual platform. Existing cybersecurity research in health care places emphasis on the protection of medical devices [13-17] and medical data [18], such as data encryption mechanisms [13], combined or not with scrambling techniques [18], managing shared health data [14], securing digital patient profiles [14], and authentication protocols for wearable devices [15]. However, this focus disregards the HIS as a holistic system, which can potentially exhibit vulnerabilities in other functions. In addition, such studies typically do not examine how potential attackers can breach the security of HISs and access, for example, ECG records, that is, other records besides those that are strictly patient focused. In this study, we address this shortcoming by providing an approach that considers and approaches an HIS as a holistic system, whereby the novelty of the AI-driven ethical hacking approach is combined with the familiar NIST framework [38], which we adapted to perform ethical hacking systematically.

Our study further contributes to the ethical hacking methods section by proposing and validating a novel AI-based ethical hacking method that incorporates optimizing and controlling modules. Several ethical hacking methods exist today, including the NIST ethical hacking framework [38], PTES, and OWASP. However, they all have limitations. For example, Nettacker, a solution developed by OWASP, contains an optimizing module, but it is not as mature, not fully published, and lacks the exploiting and controlling module. The NIST ethical hacking framework and PTES do not have optimized and controlled modules.

Our study also addressed some of the shortcomings of mainstream AI-based ethical hacking methods. Mainstream methods typically adopt reinforcement learning. Reinforcement learning is an area of machine learning concerned with how intelligent agents ought to take action in an environment to maximize the notion of cumulative rewards. This approach differs from supervised and unsupervised learning because reinforcement learning aims to learn the algorithm to obtain the best results in highly complex and uncertain situations [48]. However, as previously explained, these methods have not yet been integrated into any ethical hacking methods, and reinforcement learning itself has considerable disadvantages when applied to ethical hacking, owing to its requirement for large data sets, the lack of reliability and predictability (which could cause severe problems for the whole system), low reproducibility, and sensitivity to environmental changes [20]. The use of ACO in our optimizing module addresses these shortcomings. Our implementation of the ACO algorithm as part of the optimization module shows that it can support the conduct of an efficient vulnerability analysis and detection and offers superior results.

Our proposed AI-based ethical hacking method has practical implications, as it addresses the weaknesses of ethical hacking tools such as Nettacker, APT2, and Autosploit [40], which are used by cybersecurity practitioners. For example, Nettacker lacks an exploit module. This means that a given user will have to select the exploit tools and payload on their own, which can be challenging for nonexperts in cybersecurity. APT2, the solution offered by the Massachusetts Institute of Technology, uses Nmap to scan information; however, it lacks an optimization module. This finding suggests that the accuracy and efficiency of ethical hacking risks are inferior. Similar to APT2, Autosploit [40] is a solution that combines Shodan, Censys, Zoomeye, and Metasploit, but it does not have an optimization module. The Metasploit can then run the exploit automatically. However, similar to APT2, Autosploit risks having less accuracy and efficacy because it cannot be optimized. Currently, it is unfeasible to test all possible system configurations.

Our proposed approach addresses these limitations. The combined effect of the 2 new modules is that our approach proposes an intelligent and maintainable ethical hacking solution. First, the incorporation of the optimization module supports the identification of the shortest path for the attack, which improves the efficiency of ethical hacking. Second, incorporating the control module provides a user interface and coordinates the other modules so that ethical hacking can be carried out by nonexperts, addressing the challenge of the shortage of security experts in the health care domain.

### Limitations and Future Work

One limitation is that the simulation environment is set up in a virtual environment; although it is portable, it can potentially affect the performance of ethical hacking. As we are running the optimized and unoptimized ethical hacking methods in the same simulation environment, we would assume that this will have a limited impact on the comparable experimental results. Another limitation is that ethical hacking is set up in a network with one system or machine in the simulation environment. In real-world practice, it would be ideal to set up a network with multiple connected machines, so that ethical hacking can target multiple systems or machines.

From a cybersecurity defense perspective, future work should consider applying advanced AI techniques in HISs and explore security defense strategies to counteract cyberattacks. For example, future work could consider exploring other AI algorithms that have been used to resolve the TSP problem (eg, genetic algorithms) in the context of optimizing attack paths in ethical hacking. Future studies could also consider integrating advanced security defense strategies, such as Security Information and Event Management, Orchestration Automation and Response [49], and security operations centers. From an HIS perspective, future research could focus on building a more mature HIS that integrates diagnostic components such as arrhythmia detection and classification in ambulatory ECGs [50]. Finally, future research could expand the data set to include data from different medical devices, such as magnetocardiogram and magnetic resonance imaging.

### Conclusions

In this study, we proposed a novel AI-based ethical hacking method, which we validated using an HIS simulation platform using OpenEMR as the focal HIS. We incorporated 2 new modules into the NIST ethical hacking framework, namely the optimization and control modules, and demonstrated the ethical hacking of the HIS simulation environment using optimized (AI-based) and unoptimized methods. The results show that the optimized ethical hacking method outperforms the unoptimized method in terms of average time used, the average success rate of exploitation, the number of exploits launched, and the number of successful exploits. We were able to identify the successful attack paths and exploits. Theoretically, the findings contribute to HIS literature, ethical hacking methodology and mainstream AI-based ethical hacking method as they address some key weaknesses of these research fields. Practically, these findings have great significance for the health care sector, as OpenEMR is widely adopted by health care organizations. It also addresses some of the key weaknesses of ethical testing tools used by practitioners.

## Abbreviations

ACO: ant colony optimization
AI: artificial intelligence
ECG: electrocardiogram
HIS: health information system
NHS: National Health Service
NIST: National Institute of Standards and Technology
Nmap: Network Mapper
OpenEMR: open-source electronic medical record
OWASP: Open Web Application Security Project
PTES: Penetration Testing Execution Standard
TSP: traveling salesman problem
WHO: World Health Organization

## Data Availability

The data sets generated and analyzed during this study are available from the corresponding author upon reasonable request.
